# Ultrasound-Guided Fascia Iliaca Block Versus Pericapsular Nerve Group Block Before Positioning for Spinal Anesthesia in Patients Undergoing Surgery for Neck of Femur Fracture: A Comparative Study

**DOI:** 10.7759/cureus.68173

**Published:** 2024-08-30

**Authors:** Sandip Baheti, Mounika Yerramshetty

**Affiliations:** 1 Anaesthesiology, Dr. DY Patil Medical College, Hospital and Research Center, Dr DY Patil Vidyapeeth, Pune, IND

**Keywords:** hemodynamic stability, vas score, nerve block, spinal anesthesia, ultrasound

## Abstract

Introduction

Positioning patients with femur fractures for spinal anesthesia can be challenging due to pain. Regional anesthesia techniques, such as the fascia iliaca compartment block (FICB) and pericapsular nerve group block (PENG), have facilitated patient positioning and improved analgesia. This study compared the efficacy of ultrasound-guided FICB and PENG for pain management during the positioning of the patient for spinal anesthesia in neck of femur fracture surgeries.

Aim of the study

Ultrasound-guided fascia iliaca compartment block versus pericapsular nerve group block before positioning for spinal anesthesia in the neck of femur fracture surgeries.

Materials and methods

This prospective, randomized, single-blinded, and comparative study was conducted at Dr. DY Patil Hospital, Pune, from November 2022 to January 2024 and included 60 patients with neck and femur fractures scheduled for surgery under spinal anesthesia. Patients were randomly assigned to receive either ultrasound-guided FICB (n = 30) or PENG (n = 30) with 0.25% 20 ml of bupivacaine before positioning for spinal anesthesia. The primary outcome was to assess the Visual Analog Scale (VAS) score for pain before and after the block. Secondary outcomes included assessment of hemodynamic parameters, patient satisfaction, and adverse effects.

Results

The number of days since fracture in FICB was 2.73±0.98 and in PENG was 3.37±1.9 was comparable with no significant difference between them (p-value =0.11). The mean VAS score after the block was significantly lower in the PENG group compared to the FICB group (3.33±1.73 vs. 4.43±1.3, p = 0.007), indicating better pain relief with PENG. Both techniques were comparable in terms of hemodynamic stability. Patient satisfaction was high and similar in both groups. No significant adverse effects were reported.

Conclusion

This study observed that the ultrasound-guided pericapsular nerve group block was superior to the fascia iliaca block in providing better analgesia, good patient satisfaction, and hemodynamic stability during positioning for spinal anesthesia.

## Introduction

Pain perception is a multifaceted process that encompasses unpleasant emotional, sensory, and mental feelings triggered during surgeries [1]. This phenomenon is commonly linked to autonomic, endocrine, metabolic, physiological, and behavioral reactions. An ideal analgesic medication should offer sufficient pain relief while causing minimal surgical adverse effects [2]. Pain treatment providers face the challenge of offering in-person assistance through various methods. Fortunately, ample scientific research and clinical expertise endorse utilizing diverse technological solutions. Common pain management techniques include the use of regional anesthesia, opioids, and non-steroidal anti-inflammatory drugs (NSAIDs).

A femur fracture is a frequent orthopedic injury causing intense pain and distress due to the low pain threshold of the periosteum. Subarachnoid block (spinal anesthesia) is commonly used for femur procedures, where accurate positioning is crucial for a successful surgery. However, overriding bone in a fractured femur during movement increases discomfort and complicates proper alignment, further escalating pain and complicating the administration of a subarachnoid block [3]. Relieving pain improves patient comfort and aids in positioning for the subarachnoid block. Various intravenous drugs, including NSAIDs, opioids, ketamine, and dexmedetomidine, have been used to facilitate positioning, but these medications often cause vomiting, nausea, and respiratory depression.

The incorporation of ultrasound guidance in regional anesthetic procedures was a notable advancement. Ultrasound-guided regional anesthesia has shown improved outcomes in pain management and patient positioning. This approach allows precise localization of nerves, enhancing the effectiveness and safety of the blocks. Management of pain in femur fracture patients requires a comprehensive strategy considering both pharmaceutical interventions and regional anesthetic procedures. Ongoing research and innovation in this area are crucial to improving patient outcomes and optimizing pain management tactics.

The PENG and suprainguinal fascia iliaca blocks have become increasingly significant among regional anesthesia techniques. The PENG block, first introduced by Girón-Alango et al. [4], involves depositing the drug between the psoas muscle and the superior pubic ramus. The FICB, first described by Dalens et al. [5] in 1989, targets the femoral, obturator, and lateral cutaneous nerves, leading to their temporary loss of sensation.

However, previous studies done by Andrade et al. (2023) [6] in 384 patients who had received PENG and FICB blocks, respectively, calculated the pain scores at 6 h, 12 h, and 24 h and concluded that PENG group morphine consumption was less when compared to FICB, and more studies are required to completely comprehend the effects of this block and its advantages over the FICB block. Mosaffa et al. (2022) [7] conducted a clinical trial of 60 patients in which 30 patients received PENG and FICB, respectively, and found no statistical significance between the VAS scores (p = 0.008); however, the duration before the need for analgesic consumption after surgery was significantly extended in the PENG block group. Hence, the study revealed that PENG block is better at reducing pain due to hip fracture than FICB. However, more studies are needed to fully understand the effects of these blocks.

Therefore, my study aimed to compare the effectiveness of the pericapsular nerve group (PENG) block and the fascia iliaca (FICB) block in positioning patients with femur fractures for spinal anesthesia. We administered a 0.25% 20-ml bupivacaine injection, which is a powerful local anesthetic belonging to the amide group. It is well known for its long-lasting effects and lower side effects. Bupivacaine is a cost-effective and easily accessible option, making it a suitable choice for our comparison. This study aimed to evaluate the effectiveness of PENG and FICB blocks in providing pain relief and patient comfort during positioning the patient for spinal anesthesia and also to assess the hemodynamic parameters. The goal was to identify the superior block that can enhance the overall effectiveness of the procedure.

## Materials and methods

This prospective, randomized, single-blinded, and comparative study was conducted at Dr. D. Y. Patil Hospital, Dr. D. Y. Patil Vidyapeeth Pimpri, Pune, from November 2022 to January 2024.

The inclusion criteria consisted of ASA (American Society of Anaesthesiologists) grade I and II fit patients aged between 18 and 65 years, both male and female, with a fractured neck of the femur scheduled for surgery under the subarachnoid block and who provided informed consent.

The exclusion criteria included patients not meeting any of the inclusion criteria, those with significant neurological, cardiac, respiratory, metabolic, renal, or hepatic conditions, coagulation disorders, contraindications to spinal anesthesia or known allergies to the study medication, refusal to consent to the study, patients under 18 or over 65 years of age, patients weighing less than 40 kg or shorter than 150 cm, those with previous femoral bypass surgeries, and patients with polytrauma or infections at the injection site.

Using the mean, standard deviation, and mean difference between Group FICB and Group PENG from Kalashetty et al. [8], the data were analyzed with the WINPEPI application. With a 0.05 significance level and a power of 80%, the calculated sample size was 28 per group. To improve the reliability of the results, a total sample size of 60 was selected, with 30 participants in each group. Following written informed consent in a language comprehensible to the patients, they were allocated to one of the two groups using a computer-generated allocation table.

**Figure 1:**
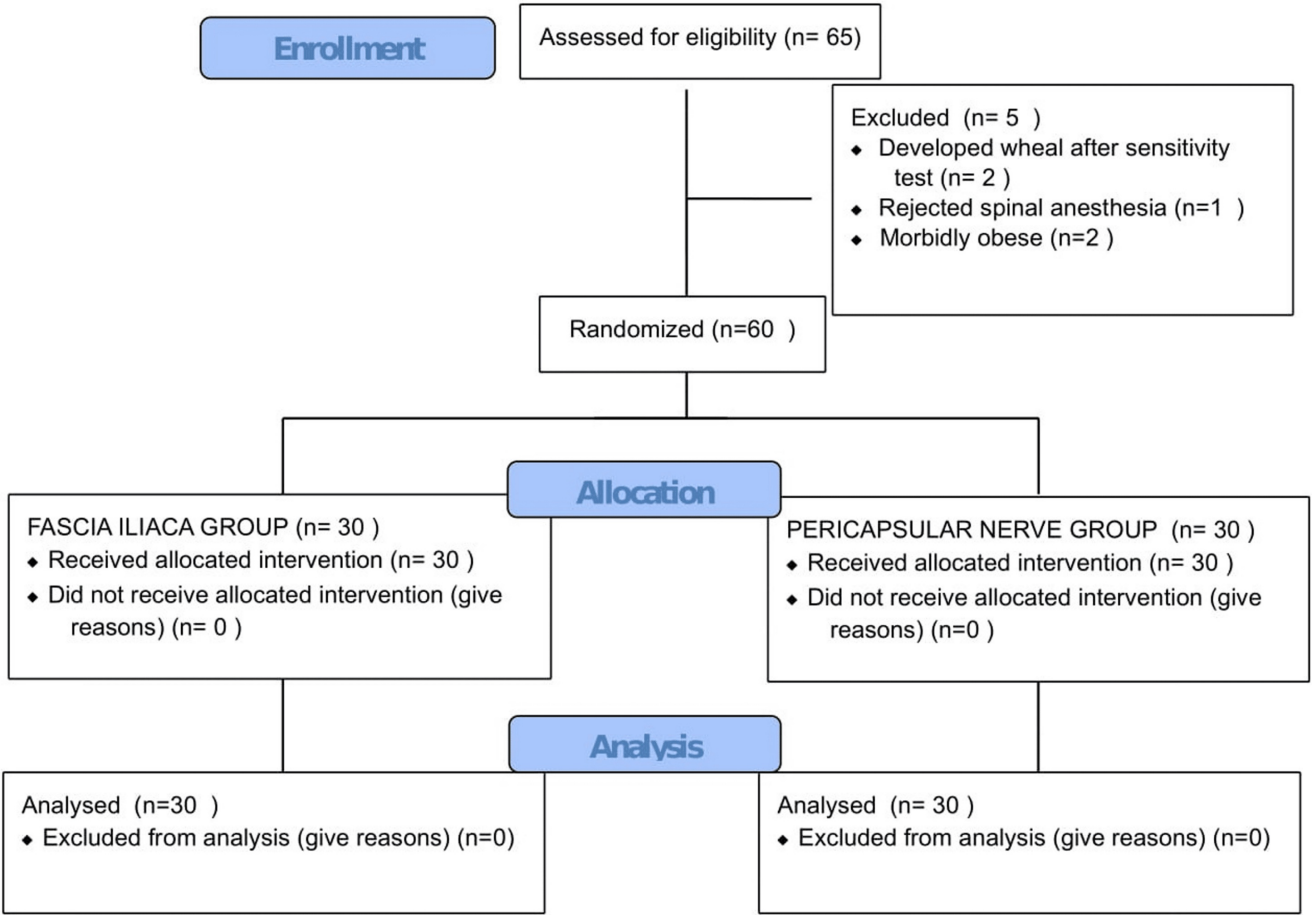
Consort Table

After randomization, two groups, Group FICB (fascia iliaca compartment block) and Group PENG (pericapsular nerve group block), were divided into 30 patients each (Figure 1).

Methodology

After obtaining informed consent, the study was conducted. A pre-anesthetic checkup was carried out the day before surgery. Before the block, basic hemodynamic parameters were noted, and the blocks were given, respectively.

**Figure 2:**
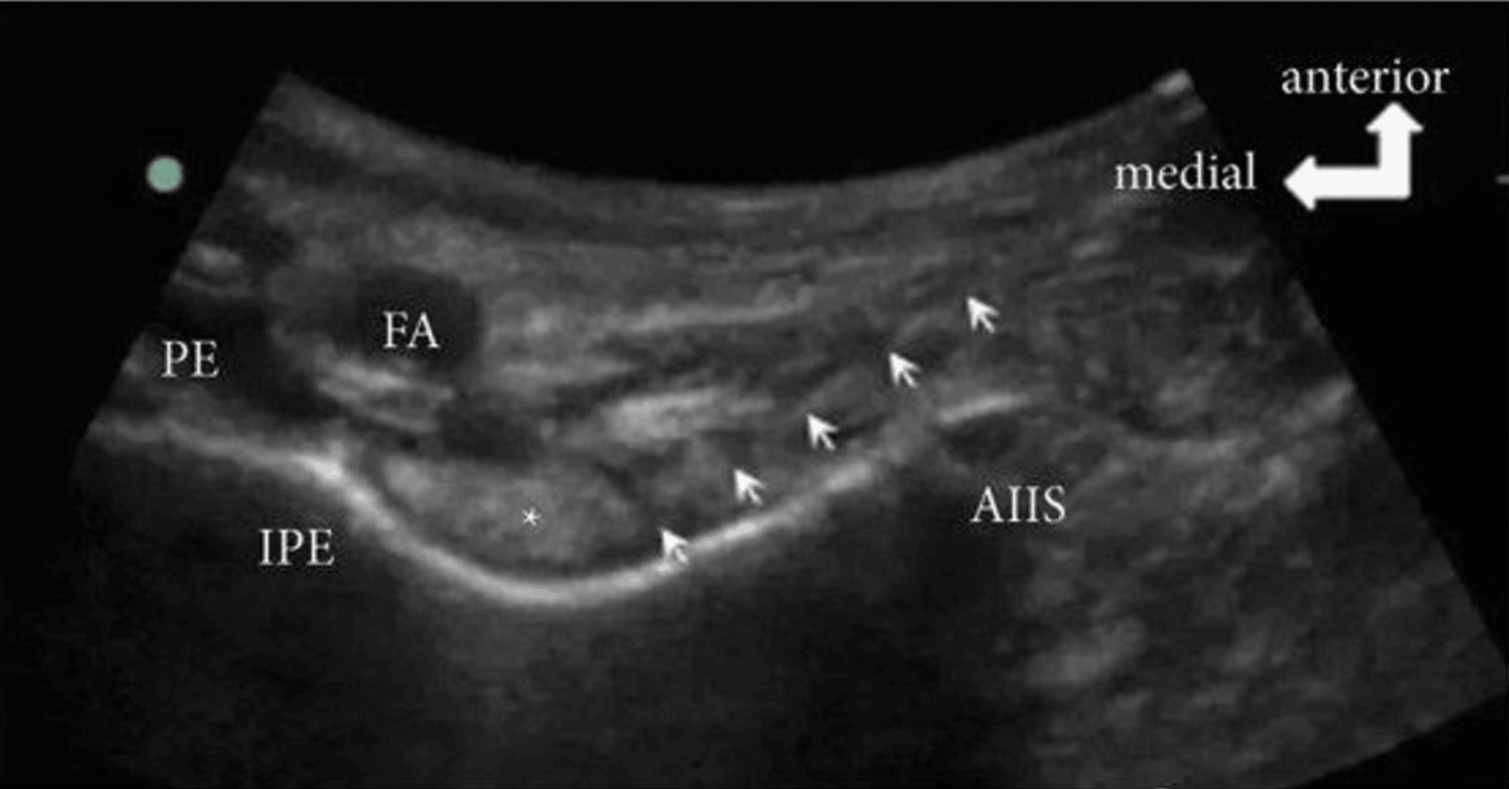
Ultrasound Image of PENG block FA: Femoral artery; PE: pectineus muscle; AIIS: anterior Inferior Iliac spine; IPE: iliopubic eminence

Group I (PENG): Following aseptic techniques and sterile precautions, a low-frequency linear ultrasound probe was placed between the anterior superior iliac spine (ASIS) and pubic tubercle, positioning the lateral margin at the ASIS. The probe was adjusted to provide a clear sonoanatomic view. The needle's entry point was chosen to enable perpendicular insertion close to the target, the ischiopubic eminence (IPE). After anesthetizing the entry point with 2 mL of 1% lidocaine, a spinal needle (23G) was inserted “out-of-plane” to reach the IPE while avoiding the femoral nerve lateral to the femoral artery. Upon contacting the bone, 20 mL of 0.25% bupivacaine was injected slowly with multiple aspirations to avoid intravascular injection and was confirmed by observing the spread of the anesthetic beneath the iliopsoas muscle (Figure 2).

**Figure 3:**
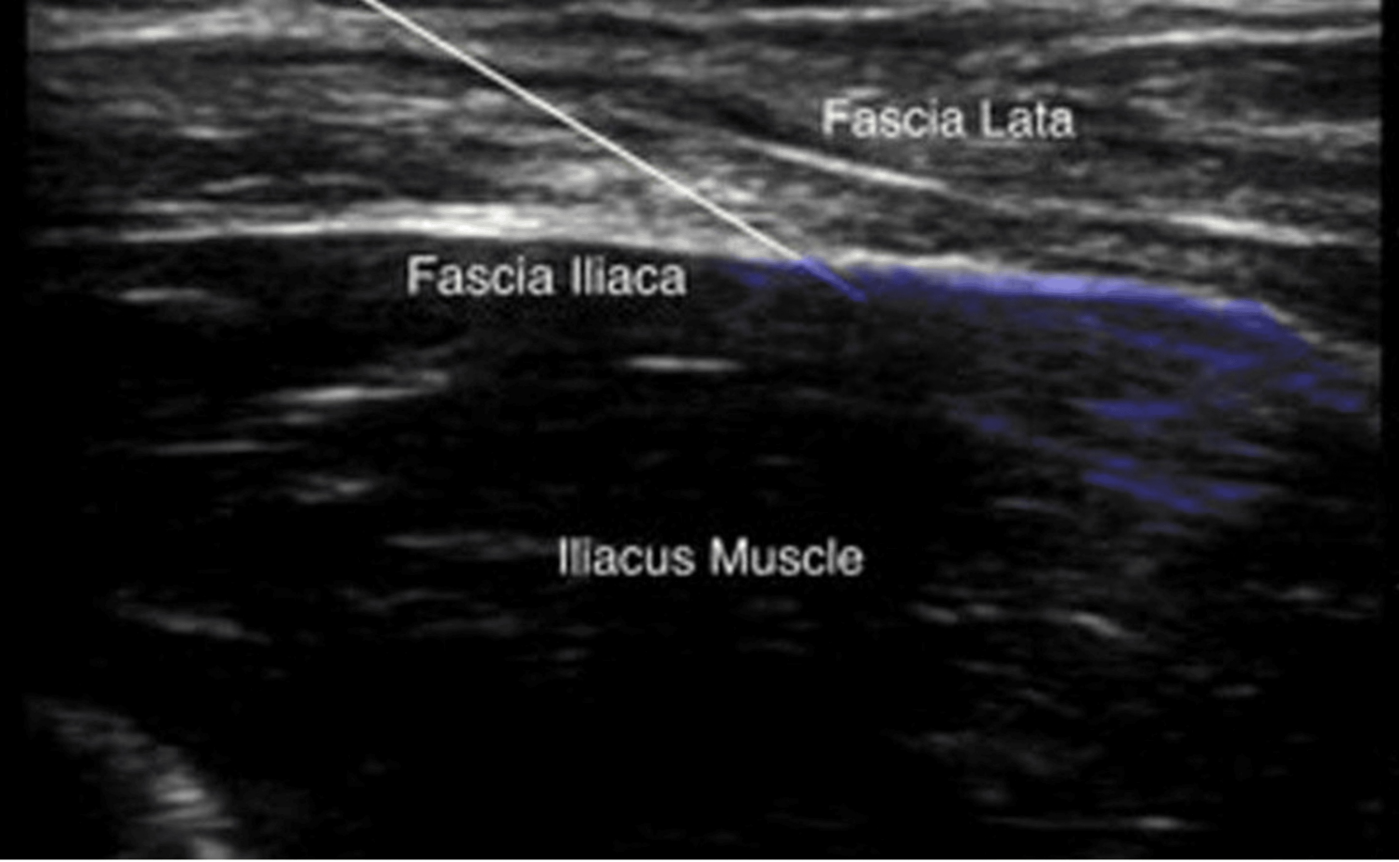
Ultrasound image of FICB block

Group 2 (FICB): Once the patient was properly positioned, under aseptic precautions, the USG probe was used to identify the femoral artery, iliopsoas muscle, and fascia iliaca. The probe was then shifted laterally to locate the sartorius muscle. The needle was inserted using an in-plane, and as it advanced through the fascia iliaca, the fascia initially indented before “snapping” back on the ultrasound image as it was pierced, producing a distinct “pop.” After confirming negative aspiration, 1-2 mL of lidocaine was injected. Subsequently, 20 mL of 0.25% bupivacaine was slowly injected with repeated aspirations. Proper needle placement was confirmed by the spread of the drug toward the femoral nerve medially and beneath the sartorius muscle laterally (Figure 3).

All the hemodynamic variables were recorded after administering the respective blocks at 5-minute intervals up to 15 minutes until positioning, which included heart rate, blood pressure, saturation, and respiratory rate. The analgesic effectiveness of each method was evaluated using the Visual Analogue Scale (VAS) score 15 minutes after the block, during patient positioning. Patient satisfaction was recorded. A subarachnoid block was administered after 15 minutes of block in the sitting position under strict aseptic conditions using a 25G Quincke needle. The block involved injecting 3 mL of 0.5% bupivacaine combined with 0.5 mL (25 mcg) of fentanyl.

Data was entered into an Excel sheet (Microsoft, Redmond, WA) and analyzed using IBM SPSS Statistics, version 20 (IBM SPSS, Armonk, NY). The results were presented in both tabular and graphical formats. For quantitative data, the mean, median, standard deviation, and ranges were calculated. Qualitative data were shown in frequencies and percentages. Independent t-test and chi-Square test were used to calculate quantitative variables and qualitative variables. A P-value of < 0.05 was considered significant.

## Results

Patients randomly received either ultrasound-guided FICB (n=30) or PENG (n=30) with 0.25% 20 ml of bupivacaine before positioning for spinal anesthesia. The study results were as follows.

Demographic profile

The distribution of age was comparable between the two groups. The majority of patients in the FICB group were aged between 61 and 70 years (33.3%), while in the PENG group, the majority were aged between 51 and 60 years (26.7%). The mean age in the FICB group was 64.3±11.85 years, and in the PENG group, it was 60.83±16.43 years. The gender distribution in the two groups was also comparable. In the FICB group, there were 53.3% females and 46.6% males, while in the PENG group, there were 36.6% females and 63.3% males. The p-value exceeded 0.05, indicating non-significance. The weight distribution in the two groups was comparable. The mean weight in the FICB group was 63.13 kg (SD 10.26), and in the PENG group, it was 62.7 kg (SD 10.17). A quantitative analysis of the mean weight among both groups revealed no statistical significance (P>0.05). The number of days since fracture in FICB was 2.73±0.98 and in PENG, it was 3.37±1.9, which was comparable with no significant difference (p-value = 0.11). (Table 1)

**Table 1:** Comparison of demographic parameters between both the groups FICB: fascia iliaca block; PENG: pericapsular nerve group block; SD: standard deviation.

Parameters	FICB	PENG	p-Value
Age (mean±SD) (years)	64.3±11.85	60.83±16.43	0.88
Weight (mean±SD) (kg)	63.13±10.26	62.7±10.17	0.87
No of days since fracture (mean±SD)	2.73±0.98	3.37±1.9	0.11

VAS scores

**Figure 4:**
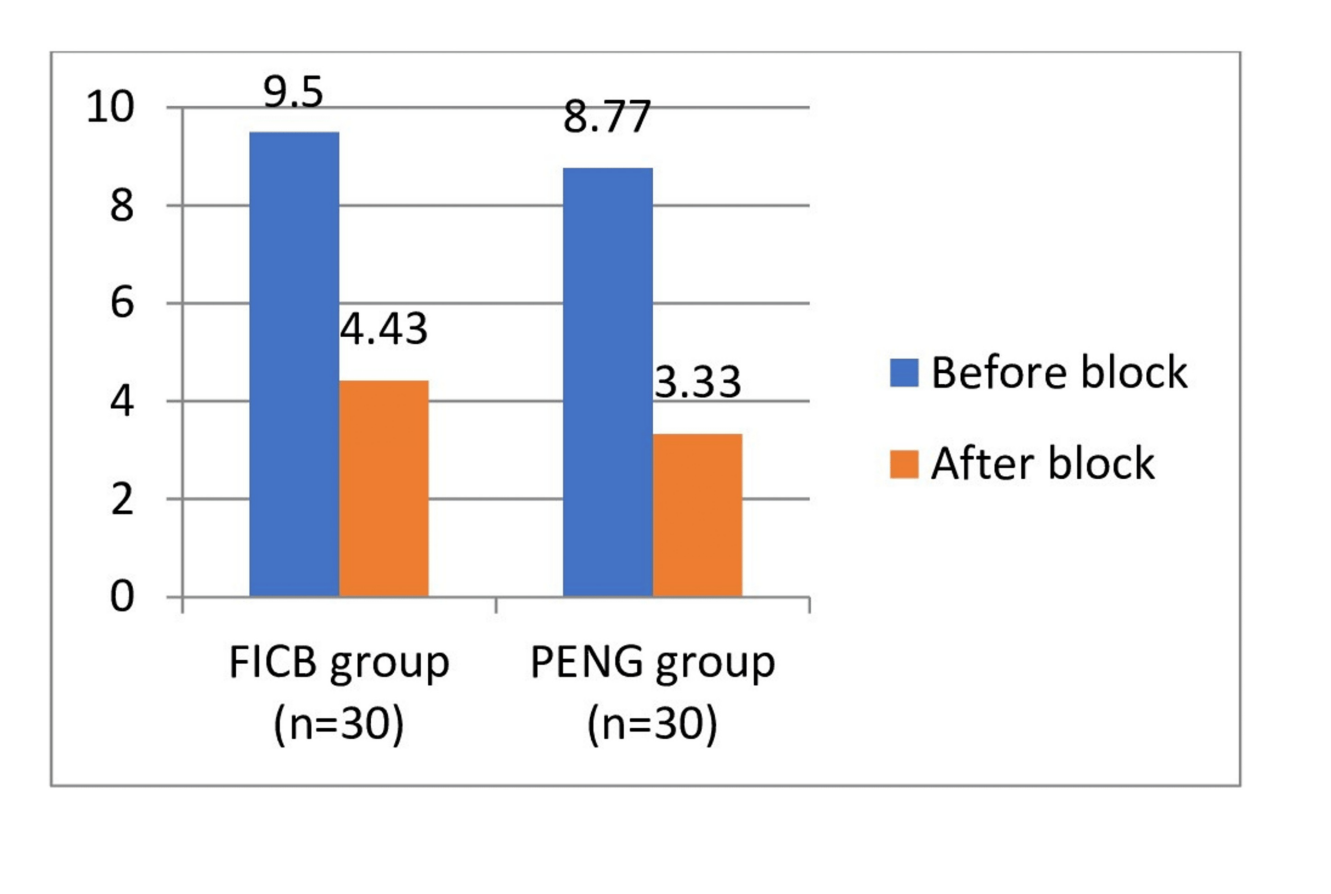
Bar Graph showing the comparison of Vas Scores before and after the blocks in both groups. FICB: fascia iliaca compartment block group; PENG: pericapsular nerve block group.

The analysis between both groups revealed that the Visual Analog Scale (VAS) score was comparable before the block. However, after the block, VAS measured at 15 minutes in the FICB group was significantly higher compared to the PENG group (4.43±1.3 versus 3.33±1.73) with a p-value<0.05, which was significant (Table 2 and Figure 4).

**Table 2:** Comparison of VAS scores before and after block between both the groups FICB: fascia iliaca compartment block group; PENG: pericapsular nerve block group; SD: standard deviation; n: number of patients; * indicates significance.

VAS	FICB group (n=30)	PENG group (n=30)	Total	p-value
Before block				
Mean ±SD	9.5±0.94	8.77±1.89	9.13±1.52	0.062
Median (25th-75th percentile)	10 (9.25-10)	9 (9-10)	10 (9-10)	
Range	7-10	2-10	2-10	
After block				
Mean ±SD	4.43±1.3	3.33±1.73	3.88±1.62	0.007*
Median (25th-75th percentile)	4 (3-5.75)	3 (2-4)	4 (3-5)	
Range	2-7	1-9	1-9	

Hemodynamic parameters

In our study, the analysis between the two groups revealed that the heart rate (HR) was comparable at baseline (82±12.46), at 5 minutes (82.38±12.34), at 10 minutes (83.18±12.83), and at 15 minutes (82.6±12.61) it was not statistically significant as the p-value exceeded 0.05. Intergroup comparison between the two groups showed that the systolic blood pressure (SBP) was comparable at 5 minutes (109.33±10.5), at 10 and 15 minutes (108±7.1, 108±8.4) (106.7±8.4, 105.33±7.7) and was not statistically significant. Similarly, intergroup comparison of both the groups showed that the diastolic blood pressure (DBP) was comparable at baseline and at 5, 10, and 15 minutes between the FICB group (79.23±9, 78.67±8.89, 80.2±9.17) and the PENG group (76.7±10.9, 77.17±9.91, 76.57±10.45), and p-value was insignificant between the groups as it was more than 0.05. Additionally, the oxygen saturation (SpO2) and respiratory rate at 5, 10, and 15 minutes were comparable between the groups with no statistical significance (p > 0.05) (Table 3).

**Table 3:** Comparison of hemodynamic parameters before and after block between both groups FICB: fascia iliaca compartment block group; PENG: pericapsular nerve block group; SD: standard deviation; SBP: systolic blood pressure; DBP: diastolic blood pressure; Spo2: saturation of oxygen; RR: respiratory rate.

Parameters		Before block	5 min	10 min	15 min
Heart rate (mean±SD)	FICB	80.03±11.25	81.87±12.45	82.77±13.27	82.43±13.53
	PENG	83.97±13.46	82.9±12.42	83.6±12.59	82.77±11.84
	p-Value (within the group)	0.224	0.749	0.8.1	0.919
SBP (mean±SD)	FICB	132.1±3.6	131.4±3.13	131.07±2.56	131±2.7
	PENG	130.87±4.8	128.7±4.10	130.2±4.54	130.6±4.87
	p-Value (within the group)	0.07	0.06	0.07	0.19
DBP (mean±SD)	FICB	79.1±5.2	77.5±4.02	76.1±3.35	76.1±3.26
	PENG	80.6±5.21	77.89±4.2	76.67±3.82	78.1±3.83
	p-Value (within the group)	0.26	0.71	0.54	0.03
Spo2 (mean±SD)	FICB	99.87±0.35	99.67±0.71	99.77±0.5	99.67±0.66
	PENG	99.87±0.35	99.63±0.67	99.67±0.61	99.67±0.61
	p-Value (within the group)	1	0.852	0.49	1
RR (mean±SD)	FICB	13.85±1.2	12.9±1.06	12.67±0.7	12.0±0.8
	PENG	14.76±1.16	13.26±0.6	12.62±0.49	12.52±0.50
	p-Value (within the group)	0.2	0.18	0.7	0.6

## Discussion

Successful neuraxial blockade depends on numerous factors, with patient positioning being critical yet often overlooked. This is particularly challenging in lower limb fractures, such as femur fractures, where discomfort and movement can exacerbate pain. Femur fractures are more prevalent among elderly patients and may present difficulties in administering spinal anesthesia due to reduced pain threshold. Spinal anesthesia is commonly preferred for orthopedic lower limb procedures. To reduce discomfort during positioning, systemic analgesics like NSAIDs, ketamine, and opioids are used, but these can cause side effects such as vomiting, nausea, and respiratory depression. Ultrasound-guided regional blocks have revolutionized pain management by providing efficient relief and simplifying subarachnoid block procedures.

As the pain intensity reduces one week after the fracture, we have excluded the patients with fractures for more than a week. The number of days since fracture in the FICB group was 2.73±0.98 and in PENG, it was 3.37±1.9 with no significant difference between the two groups (p-value =0.11).

In the current study, a comparison between the two groups revealed that the VAS score was comparable before the block between the study groups (9.5±0.94 versus 8.77±1.89). But after 15 minutes of the block, VAS in the FICB group was significantly higher compared to the PENG group when the independent student t-test was applied (4.43±1.3 versus 3.33±1.73). The p-value indicated significance between the groups, being less than 0.05.

In a study by Kalashetty et al. [8], 90 patients undergoing hip fracture surgeries under spinal anesthesia were selected to receive 20 mL of 0.25% bupivacaine in either the PENG block (n=45) or the FICB block (n=45). Both groups showed significant reductions in VAS scores at rest and passive leg raise to 15° (p < 0.0001). Thirty minutes post-block, the PENG group had VAS scores of 2.16±0.67 at rest and 3.29±0.73 with leg raise, while the FICB group had scores of 4.07±0.69 at rest and 5.11±0.71 with leg raise, indicating a significant difference (p = 0.001). The study concluded that the PENG block offers superior analgesia for hip surgery under spinal anesthesia. Unlike our study, this research measured VAS scores 30 minutes before positioning, potentially explaining the lower scores observed.

In the study by Krishnamurty et al. [9], 40 patients undergoing hip fracture surgery under spinal anesthesia were randomly assigned to either the FICB or PENG group, each receiving 25 ml of 0.25% bupivacaine. Pre-block VAS pain scores were comparable between Group P (8.4 ± 0.58) and Group F (8.1 ± 0.61) with a p-value of p = 0.9983. However, after the block (30 minutes), VAS scores were significantly lower in Group P (0.7 ± 0.2) than in Group F (3.1 ± 1.2). The study concluded that the PENG block offers superior analgesia for positioning. Unlike our study, this one measured VAS scores 30 minutes post-block and used a higher drug dose, leading to lower VAS scores.

In a study by Mariem Keskes et al. [10], patients with hip fractures received 10 ml of 0.25% bupivacaine and 10 ml of 2% lidocaine in either a PENG block or a suprainguinal fascial iliaca block (SI-FICB). Both groups showed significant pain reduction 20 minutes post-block, at rest, and while positioning for spinal anesthesia. The VAS scores dropped from 6.11 ± 0.92 to 0.61 ± 0.7 at rest and from 8.64 ± 0.83 to 1.82 ± 0.582 during mobilization in the PENG group. In the SI-FICB group, VAS scores decreased from 5.84 ± 0.82 to 0.84 ± 0.7 at rest and from 8.24 ± 0.98 to 2.16 ± 0.824 during mobilization. While the VAS scores at rest were not significantly different between the groups (p=0.078), positioning scores were significantly lower in the PENG group (p=0.046). The study concluded that the PENG block provided superior analgesia during positioning. The greater reduction in VAS scores compared to our study may be due to the addition of lidocaine and the longer post-block measurement time.

Limitations

The limitation of this study is its small sample size. Further research involving larger sample sizes and varied patient populations is necessary to confirm these findings, as pain levels can differ depending on individual pain thresholds. More research is needed on the use of fast-acting local anesthetic agents to reduce the waiting time and provide quicker pain relief for patients. Additional research is needed to assess analgesia at various time durations for a longer time span before giving a position.

## Conclusions

This study found that the ultrasound-guided pericapsular nerve group (PENG) block demonstrated greater efficacy than the fascia iliaca (FICB) block in several key aspects. The PENG block provided superior analgesia, which means it was more effective at managing pain during the procedure. Additionally, the PENG block was associated with better hemodynamic stability. This suggests that patients who received the PENG block experienced fewer fluctuations in their vital signs, such as blood pressure and heart rate, than those who received the FICB block. 

In summary, the study highlights the advantages of the PENG block over the FICB block in terms of pain relief and maintaining better hemodynamic stability, making it a preferable choice for managing analgesia during spinal anesthesia positioning.
